# Mr. Jenkinson, on Fowler's Mineral Solution

**Published:** 1807-09

**Authors:** John Jenkinson

**Affiliations:** Oxford


### *To the Editors of the Medical and Physical Journal.*

GENTLEMEN,

I Take the liberty of transmitting you a small Collection of "Facts, determining the efficacy of Fowler's Mineral Solution in Chronic Rheumatism," which was circulated about a year and a half ago amongst the Physicians, Surgeons, and Apothecaries of Manchester and its neighbourhood. The first of those facts occurred nearly four years since, and was not long afterwards published in your Journal;* it forms, I presume, the basis upon which the present use of arsenic in that disease rests, whoever may or may not chuse ingenuously to acknowledge the source from which the hint was derived. When I consider the characters of the gentlemen, in different parts of the kingdom, of whose attention to the subject I have had the pleasure to be informed, I entertain great hopes that the remedy in question will soon be more generally adopted; as the weight of their recommendation, if

* See my letter, VOL. II. p. 492.

if it should prove successful in their hands, will make up at once for the lightness of my own.

Dr. Bardsley has lately published cases, which will go far to bring it into notice; for though I have not yet had the pleasure of perusing his book, I know he has reported favourably of it from an extract or two which I have seen; and I know him too well to doubt either the candour or the success of *his* communications. I have nothing to add to the testimonies already adduced upon the subject, my own opportunities of prosecuting the inquiry having ceased for some time; but I would throw out a hint or two before I conclude. A long perseverance in a moderate dose of the solution, without augmentation, would seem to promise every advantage likely to be derived from its use, without exciting those inconveniencies which, with a full and increasing dose, are occasionally sufficient to forbid a continuance of the plan. Catarrhous symptoms, rather more troublesome than usual, are easily brought on in some persons by *full* doses of this medicine; a circumstance which may very rationally beget much caution, as to the exhibition of it in patients of a phthisical constitution. There are also irritabilities about the stomach, in which it may be requisite to watch its effects with care, and to suspend its use altogether, without delay, in case of material disturbance; but it is not *all* weak or disordered stomachs that oppose its use, for I have witnessed *good* effects from it in several dyspeptic cases, which were almost unmanageable upon other plans. Nevertheless, it may be required in other patients to resort to a fuller dose, gradually carried as high as can be borne; but in my own practice I should *now* be inclined to restrict *this* employment of the medicine to cases wherein the gentler mode had previously failed of doing good. In young persons, with full habits particularly, low diet, *small doses*, and occasional cathartics, of the saline kind, are necessary to ward off heat and other febrile symptoms, in the beginning of the plan at least; and in general it may be observed, that elderly persons, or others much debilitated, bear the medicine best, and of course most frequently profit by it. Former remedies have effected very little indeed towards affording relief in "nodosities of the joints," of the painful or rheumatic sort; and if it should appear that we possess in arsenic the means of benefiting any considerable proportion of the unhappy sufferers from that most tormenting disease, the fact itself will supercede the necessity of other eulogies. I should suppose it would be sufficient, for the purposes of truth, to refer in some part of your Journal to the letters which compose the collection I have sent you a copy of, as, with the exception of the last, they were taken from your pages; but if you should think farther notice called for by any circumstances within *your* observation, you are at liberty of course to act upon your own opinion.*

*Oxford, June* 8, 1807.

---

I Have lately had the gratificatien to learn from several quarters of the highest respectability, that Fowler's Mineral Solution has been found essentially servicable in tic douloureux, where the pain has had either regular or irregular remissions of some duration; it should be observed, that this remedy seems to require such cessations to ensure its efficacy, and that the more periodical these cessations are, the more successful it is, not only in tic douloureux, but for the most part in the other complaints which it relieves. It was originally in my contemplation to name tic dolonreux as one of the disorders in which it would be right to put arsenic prepared thus, or in some other manner,† to the test of experience, after I had ascertained its utility in chronic rheumatism; and I was only deterred from doing so by the general hopelessness of those inveterate cases. Its similarity to rheumatic affections, however, very easily and naturally pointed out tic doloureux, as requiring such a trial of the powers of the mineral solution, and I am extremely happy to learn how favourable the issue of it has proved. I hope, that the gentlemen who have ascertained the fact of its *efficacy* in that disorder will favour the public with some account of their success: the names of those to whom I allude, are such as would establish the remedy in the public opinion most satisfactorily. At this period, when the efficacy of this medicine, in the worst rheumatic cases, has been so fully proved,‡ I scarcely know any idiopathic *external* pain not connected with inflammation, and not absolutely *incessant,* in which I should not expect some *alleviation,* at least from its use, if more common remedies demanded the experiment; and perhaps it is not too much to say, that its various properties entitle it to *trial* even in other asthenic

---

* Vid. Med. & Phys. Journ. Vol. xiii. p. 16, and 525.

† The Kali Arsenicatum of St Bartholomew's Hospital Pharmacopœia is essentially the same, and a very convenient form for watery solutions.

‡ Particularly by Dr. Bardsley in his recent volume.

asthenic diseases, whether of a spasmodic nature or not, for which we are at present unprovided with efficient remedies.

Under such circumstances, little benefit would more than recompense our attention; and how is physic to be improved if new articles are not to be cautiously tried after the failure of all the old ones, whatever may be the chances against them *before hand*? Of course, I would except such diseases as originally affect the thoracic and abdominal viscera, and the lungs particularly. I have seen cancerous cases, in which it has proved the best anodyne internally employed; and athritic ones, in which it has afforded complete relief *indirectly*, by converting lingering and irregular attacks into regular paroxysms.

I am, &c.

JOHN JENKINSON.

*Oxford, July* 8, 1807.

